# The Lifted Veil of Uncommon EGFR Mutation p.L747P in Non-Small Cell Lung Cancer: Molecular Feature and Targeting Sensitivity to Tyrosine Kinase Inhibitors

**DOI:** 10.3389/fonc.2022.843299

**Published:** 2022-02-11

**Authors:** Guangjian Yang, Chengming Liu, Jiaqi Hu, Yang Sun, Peizeng Hu, Liu Liu, Haiyan Xu, Dazhou Li, Weihua Li, Yaning Yang, Nan Sun, Jie He, Yan Wang

**Affiliations:** ^1^ Department of Medical Oncology, National Cancer Center/National Clinical Research Center for Cancer/Cancer Hospital, Chinese Academy of Medical Sciences and Peking Union Medical College, Beijing, China; ^2^ Department of Thoracic Surgery, National Cancer Center/National Clinical Research Center for Cancer/Cancer Hospital, Chinese Academy of Medical Sciences and Peking Union Medical College, Beijing, China; ^3^ Drug Discovery Business Unit, PharmaBlock Sciences (Nanjing), Inc., Nanjing, China; ^4^ Department of Traditional Chinese Medicine, Yidu Central Hospital of Weifang, Qingzhou, China; ^5^ Department of Comprehensive Oncology, National Cancer Center/National Clinical Research Center for Cancer/Cancer Hospital, Chinese Academy of Medical Sciences and Peking Union Medical College, Beijing, China; ^6^ College of Computer Science and Technology, Shenyang University of Chemical Technology, Shenyang, China; ^7^ Department of Pathology, National Cancer Center/National Clinical Research Center for Cancer/Cancer Hospital, Chinese Academy of Medical Sciences and Peking Union Medical College, Beijing, China

**Keywords:** EGFR, tyrosine kinase inhibitor, molecular feature, targeting sensitivity, p.L747P mutation, non-small cell lung cancer

## Abstract

**Objectives:**

The uncommon p.L747P mutation in epidermal growth factor receptor (EGFR) exon 19 reveals to alter the response to tyrosine kinase inhibitors (TKIs) in patients diagnosed with advanced non-small cell lung cancer (NSCLC). However, the underlying mechanism is still not clear. This study aimed to investigate the clinical outcomes, binding affinities, and modes of action of currently available EGFR TKIs towards p.L747P mutation.

**Materials and Methods:**

Clinical data of NSCLC patients harboring p.L747P mutation who had received different generations of EGFR TKIs were collected from medical records. Computational structure of p.L747P was constructed and *in vitro* cellular kinase inhibition assay and mice xenograft experiment were performed to predict and confirm the binding affinities and antitumor activities of diverse EGFR TKIs.

**Results:**

A total of five metastatic NSCLC patients with p.L747P mutation were included in the final analysis. Patients treated with second-generation (2G) TKI afatinib achieved numerically longer progression-free survival (range 2.4-8.5 months) than that with first-generation (1G, range 1.4-5.5 months) or third-generation (3G, range 1.6-7.5 months) TKIs. None of the patients administered 1G or 3G TKIs achieved tumor response, but two-thirds of them treated with afatinib achieved partial response. Dynamics simulation predicted that 2G TKIs presented the best binding affinity to p.L747P mutation. The cellular kinase inhibition assay and mice xenograft experiment confirmed that afatinib could potently inhibit p.L747P-mutant cells and significantly reduce p.L747P-mutant tumor growth (*P*< 0.001), together with reduced phosphorylation of EGFR and its downstream signalings.

**Conclusions:**

The uncommon p.L747P mutation in EGFR exon 19 resulted in a poor response to first-generation EGFR TKIs. Afatinib revealed a better clinical response and binding affinity compared with osimertinib for this specific alteration.

## Introduction

Lung cancer is the leading cause of cancer-related mortality worldwide. New strategies have been developed to target specific alterations in lung cancer in the last decade and hence improved treatment outcomes and survival (1). Classic activating mutations of the epidermal growth factor receptor (EGFR) in non-small cell lung cancer (NSCLC) are found in approximately 47% of patients in Asian-Pacific countries (2). Most of these mutations occur in exons 18 to 21 of EGFR gene, which encode the main EGFR tyrosine kinase binding domain (3, 4). Exon 19 deletion (19del) and exon 21 missense mutation L858R are the two most common activating forms, accounting for nearly 80% to 90% of the total EGFR mutations, which are strong predictors of favorable response to tyrosine kinase inhibitors (TKIs) and viewed as sensitizing EGFR alterations. These mutations are most commonly seen in young Asian females diagnosed with lung adenocarcinoma (LUAD) who never smoked (5–10).

A series of randomized clinical trials have confirmed that NSCLC harboring the classic EGFR mutations responded better to first-generation (1G) TKIs than conventional chemotherapy (11–16). In addition, the second-generation (2G) TKIs afatinib and dacomitinib significantly improved the progression-free survival (PFS) and overall survival (OS) in these patients (17–19). The third-generation (3G) TKI osimertinib showed a clinically meaningful improvement in the PFS over 1G TKIs in the Asian population (20). Therefore, osimertinib is currently recommended as the first-line targeted therapy for advanced NSCLC patients carrying classic EGFR mutations. Based on the awareness of necessity to qualify EGFR mutations, therapeutic approach with EGFR TKIs based on the detection of EGFR sensitizing alterations in the kinase domain has led to a dramatic shift in the treatment paradigm in advanced NSCLC, which has represented the standard of care for EGFR-mutated patients (21, 22).

Nevertheless, a spectrum of uncommon EGFR mutations such as p.G719X, p.S768I, and p.L861Q, affecting about 10% of the NSCLC population (5, 8, 23), have been reported to be more responsive to afatinib (23–25). Uncommon EGFR alterations appeared to carry heterogeneous molecular features with clinically variable responses to TKIs and shorter PFS when compared to EGFR common mutations (26). Furthermore, few details are known about the differences on TKI sensitivity among variable EGFR alteration subtypes, even though some evidence had issued their response and survival benefit to TKIs by clinical appraisal (22). Notably, quite a part of uncommon EGFR mutations are “untested” with the polymerase chain reaction (PCR)-based assay commonly used in clinical practice, together with the adequacy, quality, and heterogeneity of tumor samples in detection techniques, which results in the inaccuracy and bias in the reported incidence of less common EGFR mutations (27). Inevitably, PCR-based commercial assays could only identify “hot spots” or common mutations to predict the responses of TKIs, and are far from sensitivity for testing other uncommon mutations, which has posed significant diagnostic issues (27). Given the urgent need for more comprehensive genetic profiling in advanced NSCLC, the introduction of next generation sequencing (NGS) covering different panels in the clinical setting has significantly improved the detection frequency of uncommon EGFR alterations, and the implement of NGS testing well characterizes the accurate EGFR mutation status (28, 29).

The p.L747P missense mutation, which also occurs in exon 19 of the EGFR gene, is rarely observed in NSCLC. It occurs due to a two-base-pair (bp) mutation (c.2239_2240TT>CC) at codon 747. This causes the substitution of the amino acid proline to leucine, leading to oncogenesis in the same way as other EGFR activating alterations (30). Due to the rarity of p.L747P mutation in NSCLC, its response to different types of EGFR TKIs is unclear and controversial, and most studies suggested that it mediated intrinsic resistance to 1G TKIs while increasing the sensitivity to afatinib (30–37). However, it still remains unclear whether this mutation improves the binding affinity and responds to osimertinib. This highlights the need for further studies to understand the underlying mechanism behind the response to different generations of EGFR TKIs in NSCLC patients with p.L747P mutation.

Therefore, in this study, we aimed to conduct a retrospective cohort study to investigate the therapeutic outcomes of diverse EGFR TKIs in patients with metastatic NSCLC harboring p.L747P mutation. Our findings were compared with published evidence. Furthermore, we also constructed the three-dimensional (3D) computational modeling of p.L747P mutation to simulate its binding activities to EGFR TKIs. The antitumor activities of EGFR TKIs for p.L747P mutation were finally evaluated and confirmed through cellular kinase inhibition assay and mice xenograft experiment.

## Material and Methods

### Patients and Data Collection

All patients diagnosed with metastatic NSCLC carrying p.L747P mutation treated at the Chinese Academy of Medical Sciences (CAMS)/Cancer Hospital from 2016 to 2020 were included in this cohort study. The p.L747P mutation in this study was identified by NGS testing which was performed in institutional laboratories or qualified third-party genetic testing companies that had acquired the national quality system certification *via* formalin-fixed, paraffin-embedded tissue samples. All of the NGS testing was performed based on the Illumina sequencing system, with same detection of a protein sequence encoded by the EGFR exon 19 with a substitution of the amino acid proline to leucine at codon 747 (p.L747P) and a DNA sequence with a 2-bp cytosine substitution to thymine (c.2239_2240 TT>CC). The medical records of these patients were retrospectively reviewed, and their clinical characteristics and targeted outcomes were recorded. The last follow-up date was July 21, 2021.

### Response Assessment

The lesion size and overall disease stage at baseline were obtained through the use of computed tomography images of the chest and abdomen, brain magnetic resonance imaging, and whole-body bone scans. Tumor response to targeted therapy was evaluated after 4 weeks of TKI initiation and subsequently every 8 weeks, and presented as either complete response (CR), partial response (PR), stable disease (SD), or progressive disease (PD) according to the Response Evaluation Criteria in Solid Tumors (RECIST) version 1.1. PFS was defined by the investigators as the time from TKI initiation to the date of documented disease progression or death from any cause (whichever occurred first). The objective response rate (ORR) was the proportion of patients with at least once confirmed CR or PR. OS was defined as the time from the diagnosis of stage IV disease to death from any cause.

### Molecular Dynamics Simulation

The 3D-modeling of p.L747P was performed based on the crystal structure of the wild-type (WT) EGFR kinase domain in complex with dacomitinib, using the Schrödinger software (2020-1 Release) (PDB: 4I23). For the prediction of bioactive conformation and binding modes with EGFR TKIs (chemical structures were listed in the **Supplementary Figure**), including afatinib (BIBW2992), dacomitinib (PF299804), osimertinib (AZD9291), poziotinib (HM781-36B), and mobocertinib (TAK-788), we conducted docking simulations using the GLIDE (Schrödinger 2020-1 Release) program from Schrödinger Inc. (Portland, Oregon). The protein preparation wizard of the Maestro (Schrödinger 2020-1 Release) interface in the Schrödinger modeling package was used to prepare the protein. Compounds were constructed using the 3D-sketcher module in Maestro. The computer-based binding free energy (ΔG_bind_) was calculated with the GlideScore method and the Molecular Mechanics/Generalized Born Surface Area (MM/GBSA) method. The electrostatic energy, van der Waals action, polar solvation energy, and total residual energy contributions were also calculated by the MM/GBSA method.

### Genetically Engineered Cell Lines

A431 cells were purchased from Nanjing Cobioer biotechnology Co., Ltd. Dulbecco’s Modified Eagle Medium (DMEM), Penicillin-Streptomycin and 0.5% Trypsin-EDTA(10X) were purchased from ThermoFisher (Waltham, MA, USA). Certified Fetal Bovine Serum (FBS) was purchased from Biological Industries (BI). Corning 96 and 384-well cell culture plates were purchased from CORNING, USA. Cell-Titer Glo^®^ was purchased from Promega Corporation (Madison, WI, USA). Complementary DNA (cDNA) of p.L747P-mutant EGFR were transfected into A431 cells using Nucleofector (Lonza), followed by clone selection using puromycin. All cell lines were authenticated by western blot and drug screening. Sequencing analysis was performed to confirm the integration of p.L747P-mutant EGFR. All cell lines used in the study tested negative for mycoplasma as determined by Real-Time PCR (Takara).

### Cell Proliferation Inhibition Assay

Cell viability was assessed using the Cell Titer-Glo assay kit from Promega (Madison, WI, USA) by quantitating the adenosine triphosphate (ATP) present in the cell cultures. A431 cells were cultured in DMEM with 10% FBS and 1% penicillin-streptomycin. Exponentially growing cells were plated in a 384-well plate at a concentration of 1000 cells/ml with 20ul per well, followed by overnight incubation at 37°C, 5% CO_2_. Compounds were prepared as 12-point, 3-fold serial dilutions in dimethyl sulfoxide (DMSO), beginning at 2mM. They were further diluted 100 folds with cell culture media and 20 µL were added to each well of cell plate. The final top concentration of compound in the assay was 10uM and that of DMSO was 0.5%. The plates were then incubated for 3 days at 37°C, 5% CO_2_. Luminescence was read after 20 minutes of incubation with the SPARK multiple plate reader from TECAN (Switzerland). The half maximal inhibitory concentrations (IC_50_) of compounds inhibiting cell viability were determined using a sigmoidal dose-response model (variable slopes, four parameters) in Prism 7 (La Jolla, CA) to evaluate the inhibitory ability of compounds on the proliferation of A431 cells.

### Mice Xenograft Experiment

The LUAD sample with p.L747P mutation was obtained from one metastatic NSCLC patient from the CAMS/Cancer Hospital and was transported directly to the laboratory after tumor tissue biopsy. The tumor sample was washed twice with cold phosphate-buffered solution and minced into smaller pieces (1cm^3^) using scissors before being implanted into a four-week-old female BALB/c nude mice. All animal experiments in this study were conducted under an institutionally approved protocol of the Animal Care and Use Committee of the CAMS/Cancer Hospital. After five generations in nude mice, the mice received an oral gavage of vehicle consisting of 0.5% methylcellulose in water, afatinib (7.5mg/kg/daily), dacomitinib (10mg/kg/daily), osimertinib (25mg/kg/daily), poziotinib (0.3mg/kg/daily), and mobocertinib (7.5mg/kg/daily) for 14 days. The xenograft tumor growth and mice body weight were monitored every three days. All the mice were killed on day 15 to harvest the tumors. The xenograft tumors were fixed in 4% paraformaldehyde for 24 hours, then sliced at a thickness of 5μm for immunohistochemical (IHC) analysis. The slices were subsequently stained using an anti-rabbit p-EGFR antibody (ab40815; Abcam, Cambridge, UK), anti-rabbit p-ERK antibody (4370; Cell Signaling Technology, Danvers, MA, USA), and anti-rabbit p-AKT antibody (4060; Cell Signaling Technology, Danvers, MA, USA) as indicated by the manufacturers’ instructions.

### Statistical Analysis

Statistical analyses were performed using the SPSS software, version 20.0 (IBM Corp., Armonk, NY, USA) and the GraphPad Prism software, version 8.0 (GraphPad Software Inc., San Diego, CA, USA). The experimental data were presented as the mean ± standard deviation. The Student’s t-test was used for comparison between two groups. The two-way analysis of variance was used for comparison between multiple groups. All reported *P*-values were two-tailed, and for all analyses, a *P*-value below 0.05 was considered statistically significant unless otherwise specified.

## Results

### Patient Characteristics

A total of five patients with metastatic LUAD harboring p.L747P mutation were included in the study. The median age was 52 (range, 41-63) years. Three patients were male, and two were never smokers. All of them received first-, second-, and third-line treatment. As first-line (1L) treatment, three patients received platinum-based chemotherapy, and two patients were treated with 1G TKIs either gefitinib or icotinib. In the second-line (2L) setting, two patients were administered 1G TKIs, one patient received 2G TKI afatinib, and another patient received 3G TKI osimertinib. In addition, as third-line treatment (3L), one patient received afatinib, and the others received osimertinib.

### Treatment Response

Among the three patients receiving 1L platinum-based chemotherapy, one achieved PR with a PFS of 5.6 months, while the other two patients only achieved SD as the best response, with a PFS of 3.0 and 4.3 months. For the two patients treated with 1G TKIs in 1L, all had SD as the best response, with PFS of 3.2 and 3.4 months.

All the five patients were treated with 2L targeted therapy, and two receiving afatinib achieved PR, with a PFS of 4.7 and 8.5 months. The patient who was administered with osimertinib as 2L therapy showed best response of SD and a PFS of 7.5 months. The other two patients treated with 1G TKIs had ORR of 0, with PFS of 1.4 and 5.5 months. In the 3L setting, one case received afatinib and achieved a PFS of 2.4 months, with SD as the best response. The other four patients treated with osimertinib achieved a PFS ranging between 1.6 to 6.3 months, with ORR of 0. Up to the last follow-up, all the patients had died. The median OS was 19.7 months (95.0% CI: 18.0-21.4). The treatment responses to EGFR TKIs extracted from published studies were summarized in **Table 1**.

**Table 1:** Responses to EGFR TKIs in NSCLC patients with p.L747P mutation from published reports.

No.	Age/Sex	Ethnicity	EGFR TKI	Best response	PFS (months)	Reference
1	63/M	Taiwan/Chinese	Gefitinib	PD	0.9	(30)
2	36/M	Taiwan/Chinese	Erlotinib	PD	2.9	(30)
3	69/M	Taiwan/Chinese	Afatinib	PR	12.0	(30)
4	49/M	Taiwan/Chinese	Afatinib	PR	19.8	(30)
5	61/F	Taiwan/Chinese	Afatinib	NE	1.0	(30)
6	NA	Taiwan/Chinese	1G TKI	PD	NA	(34)
7	66/M	Chinese	Gefitinib	PD	0.5	(32)
8	54/F	Chinese	Gefitinib	PD	1.0	(35)
			Osimertinib	PD	1.0	(35)
9	76/F	Italian	Gefitinib	NE	7.0	(36)
10	61/M	Chinese	Erlotinib	PD	1.0	(31)
11	44/F	Chinese	Afatinib	SD	24.0	(37)
12	59/F	Dutch	Gefitinib	SD	6.0	(38)
13	69/F	Japanese	Gefitinib	PD	1.6	(39)
14	80/F	Chinese	Gefitinib	SD	18	(40)
15	69/F	Japanese	Gefitinib	NA	4.0	(41)
			Osimertinib	NA	4.0	(41)

F, female; M, male; NA, not available; NE, not evaluable; PD, progressive disease; PFS, progression-free survival; PR, partial response; SD, stable disease; TKI, tyrosine kinase inhibitor; 1G, first generation.

### Binding Affinity to EGFR TKIs by Dynamics Simulation

**Figure 1:**
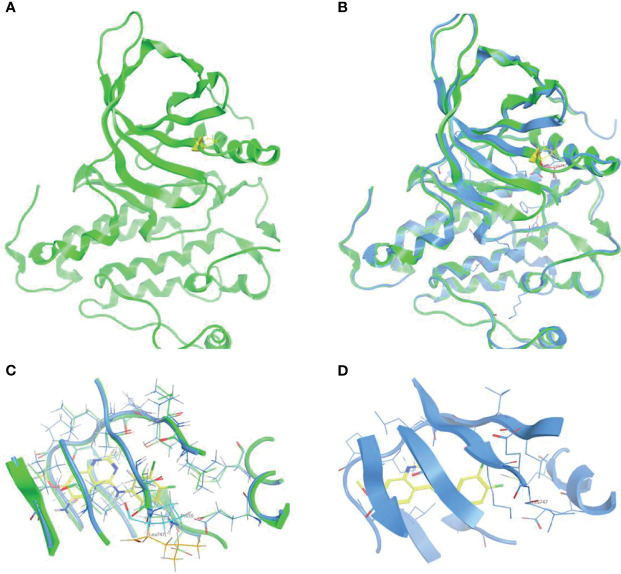
3D-modeling of p.L747P conformation **(A)** and crystal structure of EGFR wild type kinase domain in complex with dacomitinib **(B)**. ATP-binding pocket in the activating kinase domain of EGFR wild type **(C)** and p.L747P conformation **(D)**.

To elucidate the structural signature of p.L747P on the EGFR catalytic domain and investigate its affinity to currently available EGFR TKIs, 3D-modeling of p.L747P was constructed (**Figure 1A**) based on the crystal structure of the WT EGFR kinase domain in complex with dacomitinib (**Figure 1B**). The modeling revealed no significant difference in the activating kinase domain, ATP-binding site incorporating the hinge region, C-helix, P-loop, and activation loop between WT and p.L747P (**Figure 1C**). The 3D structure of p.L747P revealed that the amino acid residue leucine at codon 747 was close to the binding pocket, which was located in a key hydrophobic core that stabilized the inactive EGFR state. Compared with the WT of EGFR, no significant structural changes in the binding pocket was observed in p.L747P conformation (**Figure 1D**).

The 1G TKIs (gefitinib, erlotinib, icotinib) showed the poorest binding affinity to p.L747P mutation, with a computer-based ΔG_bind_ of -5.749 ~ -7.387 kcal/mol by GlideScore and -47.56 ~ -56.65 kcal/mol by MM/GBSA. In contrast, the 2G TKIs (afatinib, dacomitinib) conferred the best binding affinity, with a ΔG_bind_ of -7.737~ -7.953 kcal/mol by GlideScore and -61.20 ~ -65.53 kcal/mol by MM/GBSA. The 3G TKI osimertinib showed moderate binding affinity, with a ΔG_bind_ of -6.485 kcal/mol by GlideScore and -59.678 kcal/mol by MM/GBSA. These observations indicate a reduction in the binding affinity for the 1G and 3G TKIs to p.L747P when compared with 2G TKIs. In addition, we simulated the binding affinity of p.L747P with another two novel EGFR TKIs poziotinib and mobocertinib, which are designed to target EGFR exon 20 insertions under ongoing clinical trials. Dynamics simulation revealed that poziotinib and mobocertinib displayed potent and much favorable binding affinity to p.L747P mutation, with a ΔG_bind_ of -67.49~ -81.84 kcal/mol by MM/GBSA (**Table 2**).

**Table 2:** Binding free energies with different EGFR TKIs for p.L747P and WT of EGFR by dynamics calculation.

Molecule	p.L747P		WT	
	GlideScore	MM/GBSA	GlideScore	MM/GBSA
	Δ*G*_bind (kcal/mol)_	Δ*G*_bind (kcal/mol)_	Δ*G*_bind (kcal/mol)_	Δ*G*_bind (kcal/mol)_
**Gefitinib**	-5.749	-49.47	-6.291	-50.31
**Icotinib**	-6.320	-47.56	-6.174	-46.21
**Erlotinib**	-7.387	-56.65	-7.585	-57.67
**Afatinib**	-7.953	-61.20	-7.261	-58.29
**Dacomitinib**	-7.737	-65.53	-7.887	-86.24
**Osimertinib**	-6.485	-59.68	-6.170	-59.73
**Poziotinib**	-5.159	-67.49	-8.023	-94.46
**Mobocertinib**	-6.892	-81.84	-7.093	-85.55

MM/GBSA, Molecular Mechanics/Generalized Born Surface Area; WT, wild type;

ΔG_bind_, binding free energy.

**Figure 2:**
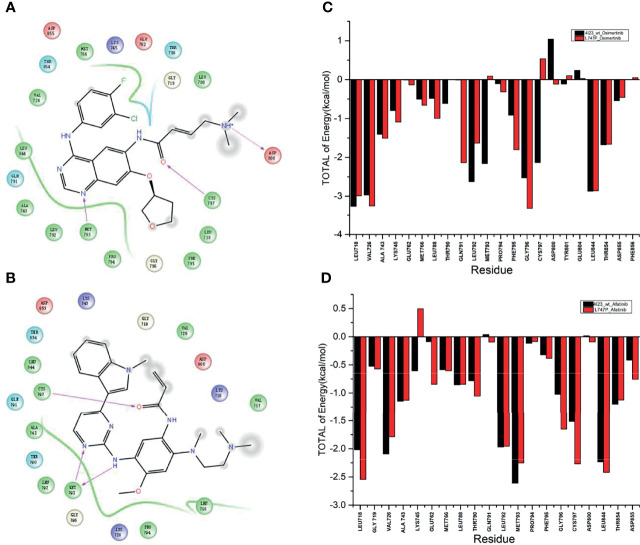
Key amino acid residues binding with molecules in the wild type of EGFR kinase domain with afatinib **(A)** and osimertinib **(B)**. Binding free energy with osimertinib **(C)** and afatinib **(D)** in EGFR wild type and p.L747P conformation.

By dynamics simulation, the binding affinity of osimertinib for p.L747P was less potent when compared with afatinib. However, the underlying mechanism for this observation has not been explored before. For this purpose, we investigated the energy contribution of residues within 4 Å of the ligand, and 10,000 conformations were extracted in 20 nanoseconds by calculation. We observed that amino acid residues that play a key role in the binding of molecules mainly were Met793 and Cys797 when afatinib (**Figure 2A**) and osimertinib (**Figure 2B**) bound with WT. The ΔG_bind_ for Met793 (-2.157 kcal/mol) and Cys797 (-2.134 kcal/mol) with osimertinib in WT was significantly lower than that for Met793 (0.091 kcal/mol) and Cys797 (0.540 kcal/mol) in p.L747P (**Figure 2C**). Conversely, the ΔG_bind_ for Met793 and Cys797 with afatinib in WT was similar to that in p.L747P (**Figure 2D**), which indicated that osimertinib was less able to bind with p.L747P compared with WT of EGFR.

**Figure 3:**
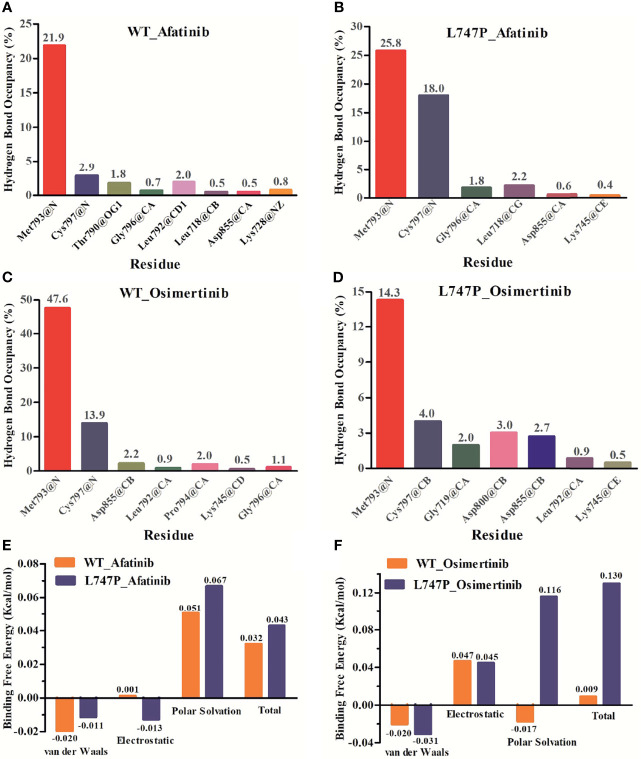
Binding affinity to afatinib in EGFR wild type **(A)** and p.L747P conformation **(B)** by hydrogen bond occupancy analysis. Binding affinity to osimertinib in EGFR wild type **(C)** and p.L747P conformation **(D)** by hydrogen bond occupancy analysis. Dynamics calculations for binding free energies with afatinib **(E)** and osimertinib **(F)** when substituting proline for leucine.

Subsequent analysis of hydrogen bond occupancy further confirmed that afatinib conferred better binding affinity to p.L747P (**Figure 3B**) than to WT of EGFR (**Figure 3A**), due to its stability in binding with amino acid residues Met793 and Cys797 to form more hydrogen bonds. However, the decreasing binding affinity of osimertinib for p.L747P may be attributed to its unstable binding mode (**Figure 3D**), along with fewer and weaker hydrogen bonds formed between residues Met793 and Cys797 than that with WT (**Figure 3C**). Molecular dynamics calculation demonstrated that the substitution of amino acid proline (0.043 kcal/mol) to leucine (0.032 kcal/mol) at codon 747 in the EGFR kinase domain had little effect on ΔG_bind_ with afatinib, resulting in an inconspicuous impact on affinity both in WT and p.L747P (**Figure 3E**). However, a distinct contribution to ΔG_bind_ was observed with osimertinib when substituting proline (0.009 kcal/mol) for leucine (0.130 kcal/mol), which eventually resulted in the weaker binding affinity to p.L747P (**Figure 3F**).

### Sensitivity to EGFR TKIs in p.L747P and EGFR WT Cell Lines

**Figure 4:**
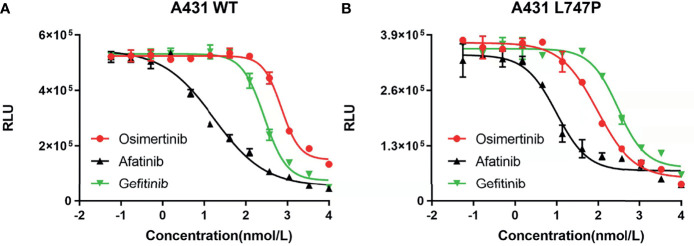
The kinase inhibition activity of 1G to 3G EGFR TKIs against EGFR wild type **(A)** and p.L747P-mutant **(B)** cell lines.

Bioluminescence technique is a rapid test for detecting cellular ATP, which is calculated as the total light emission amount-relative light unit (RLU) *via* chemiluminescence measuring devices (42). The RLU correlates with the amount and survival activity of cells, and it showed a significant decrease on afatinib both in WT (**Figure 4A**) and p.L747P-mutant A431 cell lines (**Figure 4B**), indicating that afatinib demonstrated most favorable activity for p.L747P mutation at a small concentration. In addition, the RLU did not decrease until the concentration of gefitinib elevated to 10^2^ nmol/L, which issued that it was not a sensitive inhibitor for p.L747P-mutant cells, and with poorest sensitivity to p.L747P when compared with afatinib and osimertinib. The kinase inhibition activity of diverse EGFR TKIs against p.L747P-mutant and WT cell lines was listed in **Table 3**.

**Table 3:** Kinase inhibition activity of diverse EGFR TKIs against p.L747P and EGFR WT cell lines.

Compounds IC_50_ (nmol)	A431 WT	A431 p.L747P
Gefitinib	724.8	147.3
Erlotinib	945.1	167.3
Afatinib	14.5	6.7
Dacomitinib	13.1	5.2
Osimertinib	341.6	80.9
Poziotinib	1.1	1.6
Mobocertinib	17.2	15.8

### Mice Xenograft Experiment

**Figure 5:**
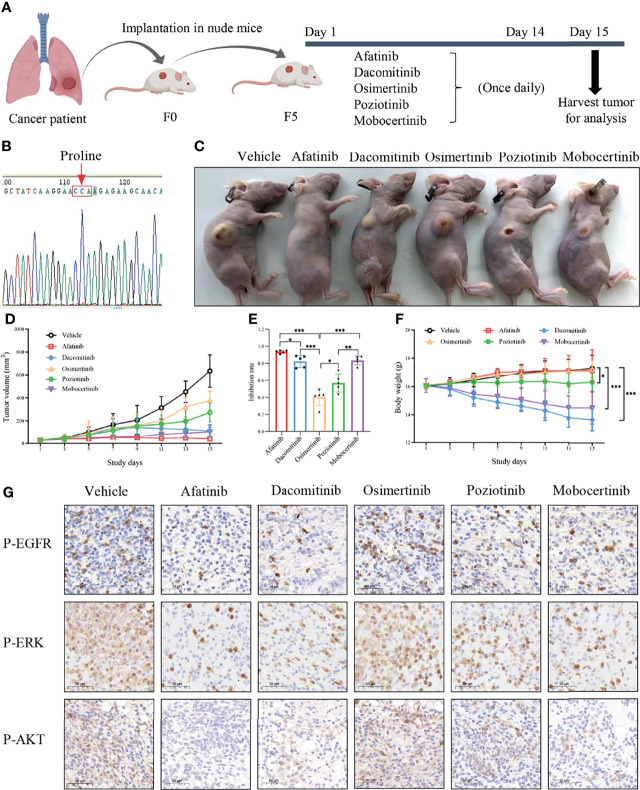
Oral gavage of vehicle, afatinib, dacomitinib, osimertinib, poziotinib, mobocertinib according to the dosing schedule **(A)** in a p.L747P-mutant patient-derived xenograft model **(B)**. EGFR TKIs for the antitumor tumor activity **(C)**, tumor volume **(D)**, tumor inhibition rate **(E)** and mice body weight **(F)** in p.L747P-mutant xenograft mouse model. Phosphorylated EGFR, and its downstream molecules phosphorylated ERK and phosphorylated AKT under inhibition of different EGFR TKIs by IHC analysis **(G)**. *P < 0.05; **P < 0.01; ***P < 0.001.

We next assessed the therapeutic efficacy of p.L747P to different EGFR TKIs in a p.L747P-mutant patient-derived xenograft (PDX) model (**Figure 5B**). After five generations in nude mice, mice received oral gavage of vehicle, afatinib, dacomitinib, osimertinib, poziotinib, mobocertinib for 14 days according to the dosing schedule (**Figure 5A**). Consistent with our findings in clinical practice, afatinib significantly attenuated both the growth and size of tumor nodules in the p.L747P-mutant xenograft mouse model when compared with the other groups (*P<*0.001, **Figures 5C-E**). Notably, dacomitinib and mobocertinib also showed a strong antitumor activity on tumor growth, but they also resulted in a significant weight reduction in the mice when compared with afatinib (*P*< 0.001, **Figures 5C-F**). As shown in **Figure 5C**, severe skin damage was found in mice treated with dacomitinib. In addition, the IHC results demonstrated that phosphorylated EGFR, ERK, and AKT were significantly decreased in tumors treated by afatinib and dacomitinib when compared with tumors treated by other EGFR TKIs. Yet, osimertinib did not effectively inhibit phosphor-EGFR and its downstream molecules (**Figure 5G**).

## Discussion

Due to the rarity of p.L747P mutation in the NSCLC population, it was not possible to accurately determine its incidence. A cohort study conducted in Taiwan, China only identified 12 patients with the uncommon p.L747P or p.L747S mutations among 2031 EGFR-mutant LUAD patients, which resulted in an overall incidence of approximately 0.59% (30). The intrinsic resistance of p.L747P mutation to EGFR TKIs was first reported in 2008 (34). The EGFR kinase 3D structure showed that condon 747 was located at the end of the β3 strand connecting to the C-helix. A cluster of hydrophobic residues contributed to the stabilization of the inactive EGFR kinase form (43).

Consistent with previously published studies, the findings from our cohort study indicated that the p.L747P mutation was associated with poor response to 1G EGFR TKIs, while a better response to 2G TKI afatinib (30–32, 34, 35, 37, 39, 41). None of the patients treated with 1G TKIs showed a tumor response, and their PFS ranged between 1.4 to 5.5 months. According to published studies, 11 patients had received 1G TKIs (gefitinib or erlotinib), and seven cases (63.6%) of them showed *de novo* resistance with PD as the best response and a PFS ranging between 0.5 to 2.9 months (30–32, 34–41). As for the 2G TKIs, most case reports and studies suggested that afatinib revealed the best activity for p.L747P, with a much longer PFS ranging between 12 to 24 months (30, 37). In our cohort study, two patients achieved PR to afatinib in 2L, with a PFS of 4.7 and 8.5 months. These findings indicated a good response to afatinib in carriers of p.L747P mutation, as also identified in the above-mentioned studies. For the 3G TKI osimertinib, case report indicated that one patient with p.L747P mutation failed to respond to it, with a PFS of only 1.0 months (29). Some small-scale studies reported moderate sensitivity to osimertinib in patients with p.L747P mutation (40, 41), yet, the evidence on the use of osimertinib to treat these patients is still insufficient. In our study, one patient received osimertinib as 2L therapy and achieved SD with a PFS of 7.5 months, and four patients were treated with osimertinib as 3L treatment and achieved a PFS ranging between 1.6 to 6.3 months with no response. However, we acknowledged that the sample size in our study was small. Therefore, further studies are warranted to investigate the real efficacy of osimertinib in carriers of p.L747P mutation.

The 3D-modeling of p.L747P constructed in our study revealed no significant difference in the activating kinase domain compared with WT of EGFR. As well, not any significant structural changes in the binding pocket was observed when substituting proline for leucine at codon 747. According to this observation, we speculated that the underlying mechanism for *de novo* drug resistance to 1G EGFR TKIs might be derived from the discrepancies in the free binding energies caused by the p.L747P conformation. As reported recently, 1G TKIs had the highest ΔG_bind_ to L747P compared with other EGFR TKIs, potentially causing binding instability and markedly decreased van der Waals interaction between EGFR tyrosine kinase and gefitinib and resulting in drug resistance (41). In addition, Ba/F3 cells expressing p.L747P mutation showed higher IC_50_ compared with the 19del and L858R mutant cells. Furthermore, immunoblot analysis has shown that p.L747P mutation was less sensitive to the 1G TKIs. In comparison, the 2G TKIs afatinib and dacomitinib could effectively inhibit phosphor-EGFR and its downstream molecules (41). Notably, dynamics simulation has shown that p.L747P mutation induced a structural change in the C-helix orientation towards the P-loop, facilitating the formation of a salt bridge between K745 and E762 residues to fix the active EGFR conformation (41). Consistent with these reported studies (30, 35, 37, 41), afatinib revealed a lower ΔG_bind_ to p.L747P and was more selective to bind with p.L747P mutation in our study when compared with osimertinib. The energy contribution simulation in our study showed that osimertinib had a significantly higher ΔG_bind_ to bind with p.L747P than that with WT of EGFR. Conversely, the ΔG_bind_ with afatinib was similar to that in WT and p.L747P. Hydrogen bond occupancy analysis further confirmed that afatinib had a better binding affinity to p.L747P due to its increasing hydrogen bonds when compared with osimertinib. All of our molecular dynamics simulation results confirmed that 2G TKIs presented the best binding affinity to p.L747P alteration.

In addition, we performed biochemical and cellular experiment to verify the mechanism of actions of gefitinib, afatinib and osimertinib targeting p.L747P mutation, and finally found that afatinib showed best binding sensitivity and antitumor activity against p.L747P-mutant cells compared with gefitinib and osimertinib. As well, the compound IC_50_ data with comparison between afatinib, gefitinib and osimertinib confirmed our findings. The mice xenograft experiment further confirmed our clinical investigation and published studies. Afatinib significantly attenuated both the growth and size of tumor nodules in the xenograft mouse model of p.L747P compared to other EGFR TKIs **(** *P<*0.001). Dacomitinib also showed strong antitumor activity on the p.L747P-mutant tumor growth, but it significantly reduced the weight of mice and caused severe skin damage compared with afatinib **(** *P<*0.001**)**. We also observed a significant reduction in the phosphorylated EGFR, ERK, and AKT in tumors treated by 2G TKIs compared with those by 1G or 3G TKIs. Interestingly, osimertinib failed to effectively inhibit phosphor-EGFR and its downstream molecules in IHC analysis, which confirmed our investigational results obtained from our cohort study and 3D-based molecular dynamics simulation. Furthermore, according to the mice xenograft experiment, mobocertinib conferred favorable antitumor activity to the p.L747P-mutant tumor. These findings were also consistent with result of binding affinity to p.L747P observed during our dynamics simulation, suggesting that mobocertinib might be a potential inhibitor for p.L747P mutation, although this agent is currently under ongoing clinical trials aiming to target EGFR exon 20 insertions.

This study has some limitations that have to be acknowledged. First, due to the scarcity and limited sample size of patients with p.L747P mutation, it is hard to conduct a prospective study enrolling enough patients. Therefore, our cohort study only included five patients with the p.L747P mutation, potentially leading to a patient selection bias even though our findings were consistent with those reported by previous studies. Furthermore, although we calculated the binding free energies of currently available EGFR TKIs by dynamics simulation to elucidate the underlying mechanism for drug resistance, exploration for molecular features and other possible signaling pathways involved in the drug resistance of p.L747P mutation is still required. Further clinical studies are warranted to confirm our findings.

In conclusion, the uncommon p.L747P mutation leads to a worse response to 1G EGFR TKIs when compared with the classic EGFR exon 19 deletions. Afatinib shows better binding affinity and antitumor activity compared with osimertinib for p.L747P mutation. NGS testing should be recommended to detect this specific mutation and hence guiding the accurate usage of TKIs in clinical practice.

## Data Availability Statement

The original contributions presented in the study are included in the article/**Supplementary Material**. Further inquiries can be directed to the corresponding author.

## Ethics Statement

The studies involving human participants were reviewed and approved by the Ethics Committee of National Cancer Center/Cancer Hospital, Chinese Academy of Medical Sciences and Peking Union Medical College. Written informed consent for participation was not required for this study in accordance with the national legislation and the institutional requirements. The animal study was reviewed and approved by the Ethics Committee of National Cancer Center/Cancer Hospital, Chinese Academy of Medical Sciences and Peking Union Medical College.

## Author Contributions

Study design and data analysis: GY, CL, and JHu. Experiment administration: JHu, CL, YS, LL, and DL. Data collection: GY, CL, PH, HX, WL, and YY. Paper writing: GY, CL, and JHu. Manuscript modification: NS, JHe, and YW. All authors contributed to the article and approved the submitted version.

## Funding

This work was supported by the Beijing Health Promotion Association (Grant No. 2021-053-ZZ).

## Conflict of Interest

Authors JH, YS, and LL were employed by PharmaBlock Sciences (Nanjing), Inc.

The remaining authors declare that the research was conducted in the absence of any commercial or financial relationships that could be construed as a potential conflict of interest.

## Publisher’s Note

All claims expressed in this article are solely those of the authors and do not necessarily represent those of their affiliated organizations, or those of the publisher, the editors and the reviewers. Any product that may be evaluated in this article, or claim that may be made by its manufacturer, is not guaranteed or endorsed by the publisher.

## Abbreviations

ATP, adenosine triphosphate; bp, base-pair; CAMS, Chinese Academy of Medical Sciences; cDNA, complementary DNA; CR, complete response; DMEM, Dulbecco’s Modified Eagle Medium; DMSO, dimethyl sulfoxide; EGFR, epidermal growth factor receptor; FBS, Fetal Bovine Serum; IC_50_, half maximal inhibitory concentration; IHC, immunohistochemical; LUAD, lung adenocarcinoma; MM/GBSA, Molecular Mechanics/Generalized Born Surface Area; NGS, next generation sequencing; NSCLC, non-small cell lung cancer; ORR, objective response rate; OS, overall survival; PCR, polymerase chain reaction; PD, progressive disease; PDX, patient-derived xenograft; PFS, progression-free survival; PR, partial response; RECIST, Response Evaluation Criteria in Solid Tumors; RLU, relative light unit; SD, stable disease; TKIs, tyrosine kinase inhibitors; WT, wild type; 1G, first-generation; 2G, second-generation; 3D, three-dimensional; 3G, third-generation; 1L, first-line; 2L, second-line; 3L, third-line; 19del, exon 19 deletion; ΔG_bind_, binding free energy.
