# Supplementary figures and images for: The Lifted Veil of Uncommon EGFR Mutation p.L747P in Non-Small Cell Lung Cancer: Molecular Feature and Targeting Sensitivity to Tyrosine Kinase Inhibitors
Source: Front Oncol. 2022 Feb 11;12:843299. doi: 10.3389/fonc.2022.843299 (PMC8873585; doi:10.3389/fonc.2022.843299)

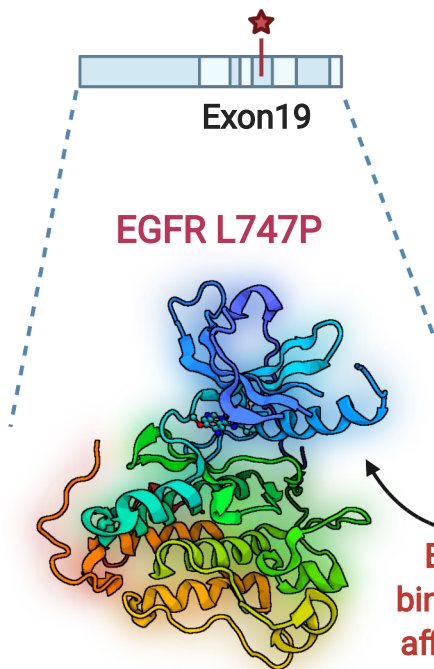

**Best  
binding  
affinity**

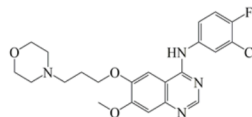

**Gefitinib**

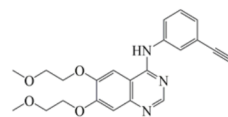

**Erlotinib**

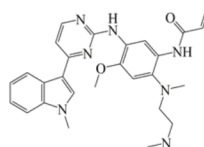

**Osimertinib**

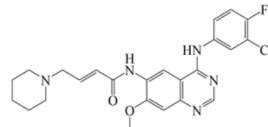

**Dacomitinib**

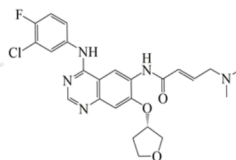

**Afatinib**

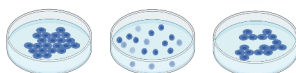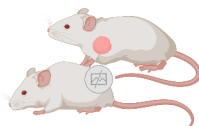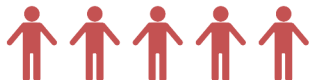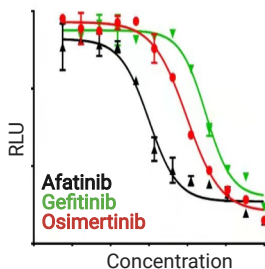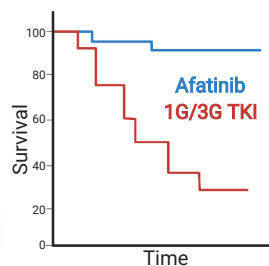

Supplement: Supplementary file 1 [file DataSheet_1.pdf]
